# Monthly Summary

**Published:** 1896-08

**Authors:** 


EDITOR THE AMERICAN JOURNAL OF DENTAL SCIENCE:

At the twenty-sixth annual meeting of the New Jersey State Denial Society, held at Asbury Park, N. J., July 29th to 31st, inclusive, the following named officers were elected:

President, Harvey Iredell, D. D. S., New Brunswick, N. J.; Vice-President, J. L. Crater, D. D. S., Orange, N. J.; Secretary, Charles A. Meeker, D. D. S., Newark, N. J.; Treasurer, Geo. C. Brown, D. D. S., Elizabeth, N. J.

CHAS. A. MEEKER, Secy.

### Monthly Summary.

SKIAGRAPHY IN DENTISTRY.—The announcement by Professor Roentgen of his new famous discovery of a method for recording upon a sensitized plate the action of radiant energy, has given to the world a new means of investigation which is already bringing forth fruit in the way of practical results. So rapidly is the improvement in these results taking place, that the work of a week ago has value only as a historical record of the steps by which greater achievements have been made.

The practical value of the X ray as a diagnostic means in general surgery and pathology became at once apparent when it was found that the bony structure of the hand could be recorded upon a sensitized plate by means of it. Its use for locating foreign bodies and in studying fractures of the bones is now clearly established as a practical method in surgical diagnosis.

The importance and value of this method as applied to dentistry it would be difficult to estimate. We present in this issue a number of half-tone reproductions of an interesting series of skiagraphs made by Professor William James Morton, which are printed in connection with his address on the subject before a recent meeting of the New York Odontological Society. It is claimed most justly by Professor Morton that any attempt at reproduction of the pictures is attended with loss of Detail, that the highest perfection of the picture is to be found only in the negative. This we can fully substantiate, for notwithstanding the fact that our illustrations represent the best that can be at present achieved by the halftone process, there is some unavoidable loss of detail as compared with the original negatives. They are, however, sufficiently clear to show what can be accomplished in the present stage of the art, and it is evident that for the diagnosis of impacted or unerupted teeth, locating the position of broken instruments in canals, detecting the projection of portions of fillings beyond the apical foramen, the diagnosis of inflammatory lesions in the jaws, necrotic bone, etc., there is a wide field of utility in dentistry for the X ray.—*The Dental Cosmos.*

---

ALLOY AND CEMENT FOR FILLING.—Procure good alloy and cement, mix the alloy as dry as will mix well; press into a flat button, the thickness of a silver dime, for an ordinary cavity. Mix the cement so as to bring it to its stickiest condition; then; the cavity having been dried and kept so, fill with the cement, and quickly, before it begins to set, press onto it a plate of amalgam about the size of face of exposed cement. Press this plate into the cavity, allowing cement to escape slightly at all parts so far as practicable. With the ball end of very small burnishers perfect the union of amalgam to the edges of the cavity, and then contour by the addition of necessary amalgam. Experience with this plan for over eleven years gives me such faith in it that I have not filled a single cavity with amalgam without the cement for five years past, and I believe the time will come when the use of amalgam without some such lining of the cavity will be considered malpractice.

This use of cement saves much discomfort to the patient from making undercuts or retaining points, also from thermal changes, and other advantages enumerated above.—W. E. Driscoll, in *Items of Interest.*

---

DR. GENESE ON AN EFFICIENT OBTUNDENT.—At the First District Dental Society of New York, Dr. Genese treated and excavated a lower first molar, capping and filling at the same time. The cavity was large and the tooth hypersensitive. Dr. Genese demonstrated the obtundent effect in this case of his preparation of carbolate of cocain, the formula of which is:

| | | |
|---|---|---|
| R. | Cocain - - - | 4 per cent. |
| | Carbolic acid - - - | 50 per cent. |
| | Benzoin gum - - | 50 per cent. |

By the aid of which he excavated the tooth painlessly.—*Cosmos.*

---

NEW TREATMENT FOR PYORRHEA.—In presenting a treatment for pyorrhea alveolaris I will not attempt to give a scientific basis for my method, since it was conceived of observation rather than a profound scientific research.

Several months ago I had occasion to see a mouth in which I had placed a piece of bridge-work three months prior. The bridge consisted of two gold caps on the lower cuspid teeth, sustaining the central and lateral incisors which, as a result ot pyorrhea alveolaris, had been lost. The cuspids to which the crowns were attached, as well as the proximal bicuspids, were also badly affected; so much so, in fact, that at the time I was apprehensive of the result. To my astonishment, three months after the operation, I found that the cuspid teeth had regained much of their firmness and an entire cessation of secretions. The bicuspids were still in the same condition as when the work was done. This caused me to suspect that the presence of the metal, which was driven well under the free margins of the gum, and made of 20k.

gold, alloyed with silver only, might account for the cure. I at once placed gold bands around the necks of the bicuspid teeth, cementing them firmly in place to prevent riding. In an incredibly short time, about these teeth, too, the flow of pus stopped. I have tried this method in three cases since with uniformly good results. In the most recent case, instead of using the gold bands, I use pure silver. This idea was suggested recently at the Johns Hopkins University, where experiments were made on silver disks by pouring pus cultures on glass slabs: where the cultures came in contact with the silver they at once became innocuous. This silver-band experiment proved by far the most valuable. In the short time of five days there was an entire abatement of the secretions. This led me to believe that it was the silver contained in the gold crown of my first experiments that did the work. The experiments at the Johns Hopkins University show silver to be the best agent of all the metals to destroy pus cultures.

The method of adjusting the bands is much the same as making a gold crown—fitting snugly to the tooth and cementing firmly. None of the metal need be exposed to view, since the only object is to have it in contact with the diseased tissue. I have not removed the bands in any of the cases, and therefore cannot state if there will be a recurrence of the trouble. I am of the opinion, however, that if all the teeth affected were treated in this manner, and the disease entirely eradicated from the mouth, there would be no recurrence. Should this not prove true the bands left on the teeth permanently would certainly be an improvement on pyorrhea alveolaris. More anon.—Dr. C. H. Rosenthal, in *Dental Registe.*

---

I am inclined to think that pressure used on one portion of a large amalgam filling, while packing it, should be very light, as heavy pressure on one part springs or bends the amalgam away from another part where it may pass unobserved. If this theory is correct, the filling will surely be disturbed many times by the occluding tooth striking it before it has hardened.

The removal of the rubber-dam, unless extreme care is exercised; will cause a shifting of some portions of the mass, owing to the tendency of amalgam to bend or spring, and thus unsuspectedly make defective edges.

The chances are that the use of a matrix would be more beneficial for this material than for any other, and it should not be removed till the amalgam is hard.—I. N. Crouse, in *Digest.*

---

LOCAL ANESTHETIC.—The following formula has been found safe and efficacious—used repeatedly without causualty or collapse; no sloughing or inflammation:

| | | |
|---|---|---|
| R. | Sulf. atrop. - - - | gr. ½. |
| | Sulf. strophanthin - - | gr. 1-5. |
| | Acid carb. (crys.) - - | gr. v. |
| | Hydrochl. cocain - - | gr. xx. |
| | Glycerin (pure) - - | fl ʒ ss. |
| | Aq., pure, *ad.* - - | ℥ ij. |

Sig.—From four to six drops hypodermically.—A. C. Hewitt, in *Register.*

---

The calculation of percentage solutions is always based on the number of grains of water in a fluid ounce. The exact weight is four hundred and fifty-five grains, and the simplest way is to multiply this number by the percentage desired. In other words, we take one grain of the drug for every hundred grains of water. Thus to obtain a four per cent solution, we multiply four hundred and fifty-five grains by four per cent, which gives eighteen and two-tenths grains, or, roughly speaking, eighteen grains to the fluid ounce of water.—*Medical Brief.*

---

AMALGAMATING GOLD SECTIONS INTO TEETH.—Coincident with the introduction of the porcelain process of filling teeth with sections of porcelain, I also practiced the art of substituting gold and other metals in the place of porcelain on the grinding surface of the teeth. When the cavity is shallow and greater toughness is required, gold will be the most admissible. The process is essentially the same as with the porcelain. Platina foil is burnished or swaged into the cavity of the tooth to secure an impression, which forms a matrix or mold; then, by fusion, gold or any other suitable metal may be run into the matrix to form a solid section or plug. When completed this corresponds to the lost portion of the tooth. The fusing of the metal may be done by means of the ordinary blow-pipe. In the course of my experiments I have found that after the gold has been molded into a section, it is advantageous to grind off the major portion of the platina lining; this exposes the surface of gold and presents a condition more favorable to unite with the amalgam. The cavity is first lined with a thin coating of amalgam, then the section is coated with a small amount. When it is ready to insert, a slight malleting will drive the section to its proper adjustment and force out surplus amalgam. Floss silk is used to tie the section in all proximal cavities and hold it while the amalgam is hardening. The section and the amalgam are then practically one compound plug. This method does away with the unsightly appearance of large blocks of amalgam, reduces the liability of shrinkage, secures the preserving qualities of the mercury as a germicide, and its ready adaptation.—C. H. Land, in *Office and Laboratory*.

---

ROOT CANAL CLEANSERS.—Take Donaldson's canal cleansers No. 5. After you have broken off the barbed portion and they appear to be worthless, smooth the end nearest the barbed portion and insert them in small wooden handles. You will find them exceedingly useful; having a fine temper they can be bent in any desired shape. Now dip them in a little sandarach varnish and wipe all excess of varnish off. Now wind a wisp of cotton around them, and they will carry any medicine to any portion of the root of a diseased tooth.

Do not wind enough cotton to fill the root tightly when using in the pumping process, especially when using peroxid of hydrogen for cleansing abscessed teeth.

The handles mentioned I make from pins secured at the butcher shop used to pin roasts with. Cut them off proper length, sandpaper and shellac them. I always put a small brass ferrule on the end nearest broach, polish it and then shellac, this gives the instrument strength and adds materially to the appearance. I also find these instruments useful in filling small canals and examining teeth for small cavities.—*Items of Interest.*

---

TREATMENT OF ROOT CANALS FOR IMMEDIATE FILLING.—Apply dam, open pulp chamber freely; soak pledget of cotton in sulfuric acid and place in pulp chamber.

1. Pump sulfuric acid into canals to apex with No. 5 Donaldson canal cleanser.

2. With drop tube flood with saturated solution bicarbonate soda. This throws out debris, neutralizes acid, and cleanses thoroughly. Dry with 5 per cent pyrozone followed by hot air.

3. Flood the canal with equal parts carbolic acid and iodin, followed by iodoform and glycerin, which force through root. Fill with chloro-percha and gutta-percha points. Fill cavity permanently.—*Items of Interest.*

---

TO BORE GLASS.—Strong glass plates are bored through by means of rotating brass tubes of the necessary diameter; which are filled with water during boring. To the water there is added finely pulverized emery. The boring cylinder is put into motion by means of a drill or bow drill. Weaker glass can be provided with holes in an easier manner by pressing a disk of wet clay on the glass and making a hole through the clay of the width desired, so that the glass is laid bare here. Then molten lead is poured into the hole, and lead and glass drop down at once. This method is based on the quick, local heating of the glass, whereby it obtains a circular crack, the outline of which corresponds to the outline of the hole made in the clay. The cutting of glass tubes, cylinders, etc., in the factories is based on the same principle.—*China, Glass and Lamps.*

THE HOUSE WE LIVE IN.—This is the advice of the late lamented Prof. J. M. Coates:

"Think deliberately of the house you live in, your body; make up your mind firmly not to abuse it, eat nothing that will hurt it; wear nothing that distorts or pains it; do not overload it with victuals or drink or work, give yourself regular and abundant sleep; keep your body warmly clad. At the first signal of danger from the thousand enemies that surround you, defend yourself. Do not take cold; guard yourself against it; if you feel the first symptoms, give yourself heroic treatment; get into a fine glow of heat by exercise; take a vigorous walk or run, then guard against a sudden attack of perspiration. This is the only body you will ever have in this world. A large share of the pleasure and pain of life will come through the use you make of it. Study deeply and diligently the structure of it, the laws that should govern it, and the pains and penalties that will surely follow a violation of every law of life or health.—*Indiana Medical Record.*

---

NERVE TENSION.—A foe to health is nerve tension, to remedy which, relaxing gymnastics can be given. By learning how to relax the muscles how to "let go," recuperation of nerve strength follows. Hurry means tension. Hurry is in the mind first, and does its most mischief there.

One may walk fast for pleasure, and not feel tired; yet, if there is hurry in the mind, to have to reach a certain place at a certain time, fatigue follows. A thought consumes more nerve force than a blow. We incapacite ourselves for the demands on us in the future, when we give way to thoughts of continued anxiety, anger, suspicion, fear, or despondency. Those of fear and despondency are especially to be avoided. Irritable moods are largely due to overstrung nerves. Relaxing exercises consume nerve force by withdrawing it from the extremities, and husbanding it at the centers. Nervousness is not always manifested by the body. When unexpressed it is of a more serious and wearing form. Continued repressed nervousness and secret worry are dangerous, on the principle of extremes meeting. Extreme tension tends to extreme prostration.

Many people wear themselves out needlessly; their conscience is a tyrant. An exaggerated sense of duty leads many a person to anxious, ceaseless activity, to be constantly doing something, over-punctual, never idle a second of time, scorn to rest; such are in unconscious nerve tension. They say they have no time to rest, they have so much to do, not thinking they are rapidly unfitting themselves for probably what would have been their best and greatest work in after years.

As there are conscious and unconscious thoughts, there are conscious and unconscious nerve tensions. Women down town shopping do not know the very tight grip they give their bundles until, on reaching home, their hands fall relaxed in their laps, and they say they are *so* tired.

Self-control of nerve force is the great lesson of health, and, therefore, of life itself. To understand how to relax is to understand how to strengthen nerves. Hearty laughter is a source of relaxation, as are also all high thoughts, as those of hope, beauty, trust or love. Relaxation is found in diversion. An occasional summer outing or holiday is necessary.—*Nashville American.*

---

THE TRUTH ABOUT BACTERIOLOGY.—During the war, when a man was wounded, the wound speedily became infested with germs and even insects, but no one thought of proclaiming that these germs produced the wound. What is true of the wound is equally applicable to other germ-producing diseases We have first the disease, which produces a suitable soil and environment, then the life germ. This is the history of life phenomena the world over.

Take phthisis for example. Consumption begins in the alimentary canal as a fermentation. This fermentation extends to the blood, which becomes disorganized. Whenever the blood contains any abnormal substance, the lymphatic system becomes involved. The lungs and mesentery are rich in lymphatic tissue, from which the disease radiates. Tubercle tissue, being poorly organized and having no function, soon breaks down, and when it does, it becomes a suitable soil for the tubercle bacillus. Whether this tubercle bacillus is the product of spontaneous generation or drawn into the lungs during respiration, is not clear. Those who claim that all life must spring from previously existing life incline to the latter view. But we really know nothing, and reason chimes in with the many eminent scientists who cling to the doctrine of spontaneous generation.

To assert that any disease is the result of germ action is putting the cart before the horse. Normal tissues offer no inducements for germ colonization. While bacteriology has led the profession into the gravest errors, we would not utterly condemn it. It may be that, in the evolution of this theory, bacteriology may prove the indirect means of bringing us nearer to the truth. All natural phenomena have their value. All are links in an endless chain of evidence. But we are too apt to consider *post hoc* the *proper hoc.* Bacteriology as the natural history of the various germ species found in the human body, products of disease, is one thing, and bacteriology in the role of pathology is quite another. Let us discriminate.—*Medical Brief.*

---

CALOMEL TREATMENT IN TYPHOID FEVER.—In the "Yale Medical Journal," Dr. Gustavus Elliot, of New Haven, Conn., gives the following summary of his treatment at the close of his article on typhoid fever.

In conclusion the following general rules should never be forgotten :

I. Keep the patient in bed until for an entire week the temperature has been normal.

II. Keep him on a sterilized liquid diet as long as he remains in bed.

III. At the beginning of the disease, give ten grains of calomel on alternate days.

IV. Give one grain of carbolic acid and three drops of tincture of iodine, every four hours, during the entire illness.—*Medical World.*

A CHEAP HYDROGEN-GAS FLASH LAMP.—Perforate the tightly fitting cork of a two-dram bottle to admit, with very close fit, an ordinary glass medicine dropper of which the taper portion has been bent at right angles and the orifice made very small by heating in Bunsen burner till it contracts a little.

In the bottle put a piece of granulated zinc the size of a pea and a little less than a dram of hydrochloric acid. Hydrogen gas will be evolved which, if ignited, will burn with intense heat as it issues from the tube. A fresh charge is required each time that it is brought into use.—W. S. Elliott, in *Items of Interest.*

---

IN treating dead teeth some dentists lay stress on the necessity of removing every vestige of the pulp. I suppose I must consider it heterodoxy, but I have often left in a portion of the pulp, depending on its harmlessness by converting it into a condensed thread of leather by tannin moistened with creasote and oil of cinnamon.—*Items of Interest.*

---

CATAPHORESIS is now the popular obtundent in dentistry. It promises to be a very serviceable method for the relief of sensitive dentine. Every operator who has a spark of manhood will hail with gladness this new Lethe.—*Western Dental Journal.*

---

IN ANESTHESIA, when chloroform or ether is used, a late method which seems reasonable, is to cocainize the mucous membrane of the nose, and thus prevent irritation to the nerves distributed to that membrane, vomiting, etc. This seems to be a very plausible method.—*Western Dental Journal.*

